# Association Between Lower Extremity Venous Insufficiency and Intrapartum Fetal Compromise: A Nationwide Cross-Sectional Study

**DOI:** 10.3389/fmed.2021.577096

**Published:** 2021-07-09

**Authors:** Ángel Asúnsolo, Chen Chaowen, Miguel A. Ortega, Santiago Coca, Luisa N. Borrell, Juan De León-Luis, Natalio García-Honduvilla, Melchor Álvarez-Mon, Julia Buján

**Affiliations:** ^1^Department of Surgery, Medical and Social Sciences, Faculty of Medicine and Health Sciences, University of Alcala, Alcalá de Henares, Spain; ^2^Ramón y Cajal Institute of Sanitary Research (IRYCIS), Madrid, Spain; ^3^Department of Epidemiology and Biostatistics, Graduate School of Public Health and Health Policy, The City University of New York, New York, NY, United States; ^4^Department of Medicine and Medical Specialities, Faculty of Medicine and Health Sciences, University of Alcalá, Alcalá de Henares, Spain; ^5^Service of Gynecology and Obstetrics, Section of Fetal Maternal Medicine, University Hospital Gregorio Marañón, Madrid, Spain; ^6^Immune System Diseases-Rheumatology and Oncology Service (CIBEREHD), University Hospital Príncipe de Asturias, Alcalá de Henares, Spain

**Keywords:** placental insufficiency, varicose veins, intrapartum fetal compromise, Spain, fetal

## Abstract

**Introduction:** Chronic venous disorder (CVeD) has a high prevalence, being commonly diagnosed by the presence of varicose veins. In fact, the development of varicose veins in lower extremities and/or pelvic venous insufficiency (LEPVI) is frequent. However, its potential impact on fetal health has not been investigated. This study aimed to examine whether the presence of varicose veins in women's LEPVI is related to an intrapartum fetal compromise event.

**Materials:** A cross-sectional, national study was conducted using medical administrative records (CMBD) of all vaginal births (*n* = 256,531) recorded in 2015 in Spain. The independent variable was defined as the presence of varicose veins in the legs, vulva, and perineum or hemorrhoids. A logistic regression model was used to assess the association of interest.

**Results:** Among women with vaginal deliveries, those with varicose veins in their LEPVI have a significantly greater odds of intrapartum fetal compromise (OR = 1.30, 99.55%CI = 1.08–1.54) than their counterparts without varicose veins. After adjustment, this association remained significant (OR = 1.25, 99.5%CI = 1.05–1.50).

**Conclusions:** Our findings of an association between varicose veins in women's lower extremities and/or pelvis and intrapartum fetal compromise suggest that varicose veins may be a novel and important clinical risk factor for fetal well-being and health.

## Introduction

Within the vascular disorders, the term chronic venous disorder (CVeD) is used to describe the spectrum of morphological and functional abnormalities of the venous system (1). One of the manifestations of CVeD is the development of varicose veins. The development of varicose veins in lower extremities and/or pelvic venous insufficiency (LEPVI) during pregnancy is a common complication (2, 3). However, its clinical relevance has not been investigated.

Recently, it was observed that LEPVI was associated with the development of structural lesions of hypoxemic pathogenesis of the placental villi (4, 5). This association between varicose veins in the infra-diaphragmatic area and placental damage could be explained by the impact of gestational venous hypertension and blood stasis in the placental area and/or abnormal placental development. Either one of these conditions could produce a high-resistance, low-flow circulation predisposing to hypoperfusion, hypoxia, reperfusion injury, and oxidative stress within the placenta. If the hypoxic insult is severe enough and long lasting under intensifying conditions such as a vaginal delivery, trophoblastic function could be altered enough to affect fetal well-being.

Uterine contractions in labor result in a 60% reduction of uteroplacental perfusion, causing transient fetal and placental hypoxia (6). A healthy term fetus with a normally developed placenta is able to accommodate this transient hypoxia. However, when there is a preexisting placental dysfunction, this dysfunction predisposes the fetus to intrapartum fetal compromise (IFC) (6). For example, placental infarct and fetal compromise could be observed in a variety of ways, such as fetal distress, passage of meconium *in utero*, or abnormal acid–base balance, and even death in extreme cases. Women with pre-labor placental dysfunction are more likely to develop IFC. However, especially in normal grown fetuses and low-risk populations, there are no tools to identify these conditions during pre-labor. Thus, the presence of varicose veins could be an external symptom and marker for this dysfunction.

This study examines whether there is an association between the presence of LEPVI and IFC in women who had vaginal deliveries in Spain in 2015.

## Methods

### Design

**Figure 1:**
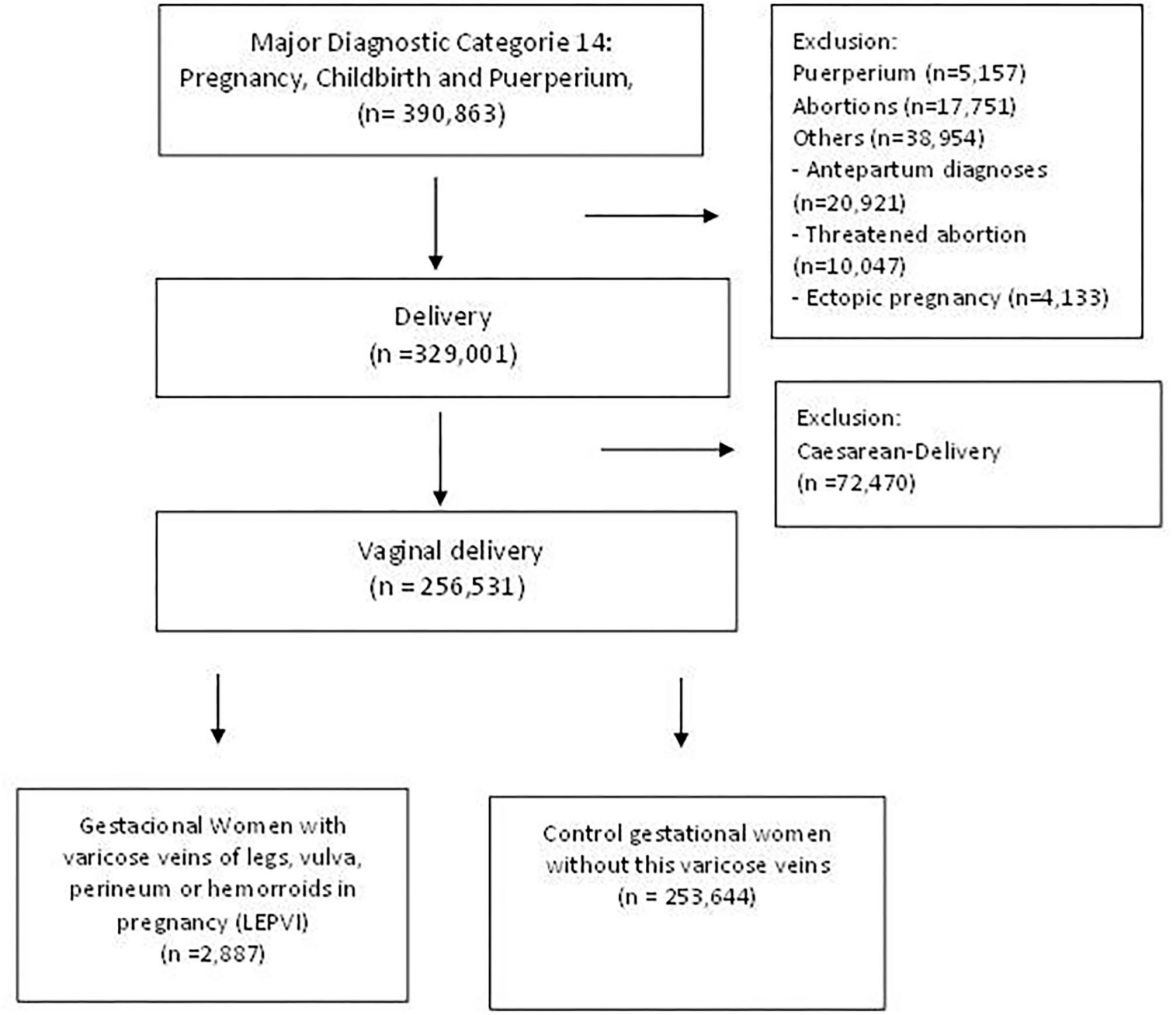
Flowchart for the study population.

A national cross-sectional study was conducted including all vaginal births (*n* = 256,531) occurring in hospitals in Spain in 2015. We selected our population using the diagnosis-related groups (DRGs), codes 372-372 and 652 (all patient refined DRGs, version 32). Please see flowchart in Figure 1 for further details on the sample size and exclusions. The Health Information Institute of the Spanish Ministry of Health provided data from the Minimal Basic Dataset (CMBD), Hospital Discharge Records in the National Health System (7). The CMBD is a mandatory and common medical administrative registry for all hospitals in Spain containing the administrative, demographic, and clinical information for users, centers, and units attending the patients and their treatments. Specifically, it contains 19 compulsory variables, with the most important being age, sex, main diagnosis, secondary diagnoses (diagnoses that coexist with the main diagnosis at the time of admission or have developed during the hospital stay), procedures, and circumstances regarding hospital discharge. The International Classification of Diseases, Ninth Revision, Clinical Modification (ICD-9 CM) codes were used for the diagnosis.

### Variables

The exposure was specified as pregnant women who presented varicose veins in the legs (ICD-9-CM: 671.0), varicose veins in the vulva and perineum (ICD-9-CM: 671.1), or hemorrhoids (ICD-9-CM: 671.8). The main outcome variable was signs of IFC due to inadequate placental function, specified as a composite variable of the presence of any of the following: fetal distress (ICD-9-CM: 656.3), abnormal acid–base balance, intrauterine acidosis or meconium in liquor (ICD-9-CM: 656.8), and placental infarct or abnormal placenta (ICD9-CM: 656.7). We considered the following variables as confounders: age, toxic habits, and the presence of comorbidities. Specifically, diagnosis of hypertension, cardiac pathology, respiratory disease, cancer, kidney or liver diseases, thyroid pathology, diabetes mellitus, obesity, and alterations in coagulation as well as the presence of depression or dementia were considered to calculate the Charlson index of comorbidity for each woman (8). A complete list of the ICD-9-CM codes used to classify the variables is provided in the Supplementary Material.

### Statistical Analysis

**Figure 2:**
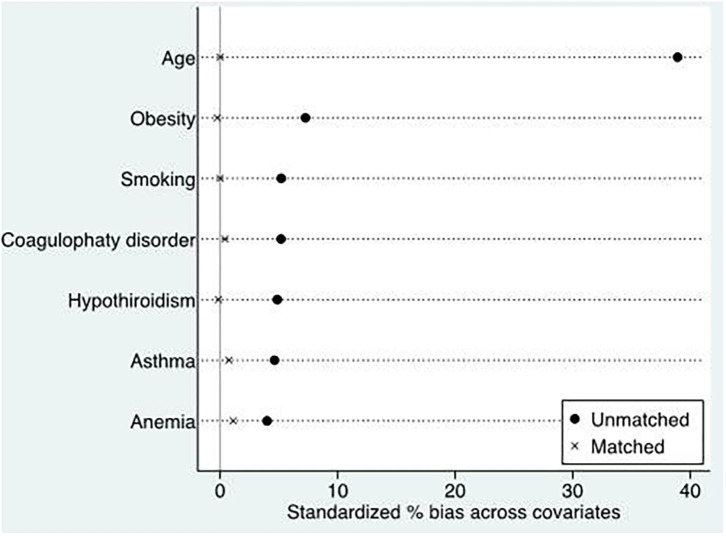
Propensity score matching of women with and without varicose veins (LEPVI).

Descriptive analysis for the population was performed presenting means and proportions according to whether the variable was continuous or categorical, respectively. A logistic regression model was used to quantify the association between the presence of LEPVI and IFC event before and after adjustment. Firstly, we fitted an explanatory logistic regression model with IFC as the dependent outcome and LEPVI as the main exposure before and after controlling for the potential confounding variables included in the database. The covariates selected had to have a plausible biological relation with the exposure and/or the outcome. Because we noted important differences in the baseline clinical characteristics between women with and without LEVPI, we also conducted a second analysis using propensity scores to mitigate confounding bias caused by the imbalance between the characteristics of the groups under comparison (Figure 2). We developed a score representing the propensity (i.e., the conditional probability) of a woman who has varicose veins in light of their clinical characteristics. We used the propensity score to join, without replacement, women with varicose veins and women without varicose veins at a 1:1 ratio. Odds ratios (ORs) were calculated using univariate conditional logistic regression. Given the number of covariates and the comparisons made, an α < 0.005 was considered as significant. Finally, we conducted a sensitivity analysis. We fitted a logistic regression model including only hospitals with more than 500 births and the presence of at least one case of LEVPI in women. STATA/IC (version 14.2) was used for all the statistical analyses.

We followed the RECORD recommendations (reporting of studies conducted using observational routinely collected data) as an extension of the existing STROBE guidelines in this paper.

This study was approved by the Clinical Research Ethics Committee of the Gómez-Ulla-UAH Defense Hospital (37/17).

## Results

The prevalence of LEPVI among pregnant women was 1.13% (*n* = 2,887; Table 1). When compared with women without LEPVI, those with LEPVI were older and more likely to smoke, to be obese, and have asthma, hypothyroidism, coagulation disorders, and anemia (all *p* < 0.05). The high prevalence for these conditions yielded a high prevalence for the Charlson Index score for women with LEPVI. There were no differences observed for hypertension and diabetes between pregnant women with and without LEVPI.

**Table 1:** Descriptive statistics for selected characteristics for pregnant women with and without LEPVI, Spain 2015.

**Characteristics**	**LEPVI % (N)**	**No LEPVI % (N)**	***P*-value**
Total	1.1 (2,887)	98.9% (253,644)	
Age, mean (SD)	33.55 (4.97)	31.49 (5.59)	<0.001
Smoking (%)	7.9 (229)	6.6 (16,812)	0.006
Obesity (%)	2,8 (80)	1.7 (4,287)	<0.001
Hypertension (%)	2.7 (77)	2.7 (6,725)	0.95
Diabetes (5)	5.7 (164)	5.3 (13,491)	0.38
Asthma (%)	2.3 (67)	1.7 (4,392)	0.015
Hypothyroidism (%)	7.7 (227)	6.5 (16,610)	0.022
Coagulopathy Disorder (%)	1.0 (29)	0.5 (1,383)	0.002
Anemia	1.9 (54)	1.4 (3,564)	0.039
**Charlson index score (%)**			
0 1 2	96.8 (2,795) 3.1 (89) 0.1 (3)	97.4 (247,163) 2.4 (6,207) 0.1 (274)	0.090

*Myocardial infarction, congestive heart failure, cerebrovascular disease, peripheral arteriovascular disorders, paralysis, renal disease, liver disease, rheumatic disease, AIDS/HIV, peptic ulcer disease excluding bleeding, depression, dementia, alcohol and drugs consumption are <1% in both groups. None of them were statistically significant. P-values were obtained from chi-squared statistics with the exception of the one for age (t-test). LEPVI, varicose veins in lower extremities and/or pelvis; N, number of patients; %, percentage; SD, standard deviation*.

Women with LEPVI were more likely to have conditions associated with IFC (9.4%) than women without LEPVI (7.4%; Table 2). Women with LEPVI have a 30% (OR = 1.30, 99.5%CI = 1.08–1.54) greater odds of having a birth outcome with IFC relative to women without LEPVI during pregnancy. Women with LEPVI also have a greater odds of placental dysfunction (OR = 1.74, 99.5% CI = 1.00–3.05) than their counterparts without LEPVI. After adjusting for age, smoking, obesity, asthma, hypothyroidism, coagulopathy disorders, and anemia, these associations remained significant but slightly attenuated for IFC [adjusted OR (aOR) = 1.25, 99.5% CI = 1.05–1.50] and placental dysfunction (aOR = 1.23, 99.5%CI = 1.01–1.49; Table 2). However, there was no association between LEPVI and fetal distress (aOR = 1.01, 99.5% CI = 0.46–2.21) after adjustment.

**Table 2:** Prevalence estimates and OR with their 99.5% confidence intervals for loss of fetal well-being among pregnant women, Spain 2015.

	**LEPVI** **% (*N*)**	**No LEPVI** **% (*N*)**	**Unadjusted OR [99.5% CI]**	**Adjusted^**a**^ OR [99.5% CI]**
Intrapartum fetal compromise	9.4 (270)	7.4 (18,792)	1.30 [1.08–1.54]	1.25 [1.05–1.50]
Fetal distress	0.5% (13)	0.4% (1,110)	1.03 [0.47–2.26]	1.01 [0.46–2.21]
Placental infarct or abnormal placenta	0.9% (26)	0.5% (1,313)	1.74 [1.00–3-05]	1.72 [0.98–3.00]
Abnormal acid-base balance, intrauterine acidosis or meconium in liquor	8.2% (236)	6.6% (16,633)	1.27 [1.05–1.54]	1.23 [1.01–1.49]

a *Adjusted for age, smoking, obesity, asthma, hypothyroidism, coagulopathy disorders, and anemia. LEPVI, varicose veins in lower extremities and/or pelvis; N, number of patients; %, percentage; CI, confidence interval*.

The ORs between LEVPI and IFC showed the same direction and remained significant regardless of the method used for the analysis. The OR based on selected hospitals with more than 500 births in 2015 and the presence of at least one case of LEPVI in women was similar to the one obtained using the entire database (aOR = 1.21, 99.5% CI = 1.01–1.46). However, the propensity score analysis yielded a greater odds of IFC for women with LEVPI (aOR = 1.84, 99.5% IC = 1.37–2.47) relative to those without LEPVI.

## Discussion

Our findings show that LEPVI may explain IFC due to a placental hypoxic dysfunction in women who give birth by vaginal delivery after controlling for age, smoking, obesity, asthma, hypothyroidism, coagulopathy disorders, and anemia. Fetoplacental circulation depends on a complex balance between arterial, placental, and maternal venous circulation. Previous studies have shown that vascular diseases (high blood pressure, heart failure, etc.) and blood disorders (anemia, clotting disorders, etc.) in the mother can cause placental insufficiency (also known as uteroplacental vascular insufficiency) and placental and fetal hypoxia (9–11). Our findings suggest that a common pathological condition such as LEPVI may be an independent risk factor for the onset of this IFC not yet identified. Therefore, varicose veins could be a novel risk factor for placental vascular alterations.

While the pathogenic mechanisms underlying the association between gestation and LEVPI with the development of IFC have not been established, it is worth noting that the development of LEPVI during pregnancy causes structural alterations in the placenta of hypoxic pathogenesis. Previous studies (4, 5) have shown a relationship between maternal venous insufficiency and placental hypoxia. In addition, histopathological Tenny–Parker changes in the villi or placental damaging alterations, increase of apoptosis, as well as changes in the gene and protein expressions of HIF-1α were observed (4, 5). Therefore, there is a biological basis for our findings. In this sense, it has been demonstrated how, in the placental villi of patients with CVeD, there is an increase in oxidative stress with an increase in lipid peroxidation levels with angiogenic and lymphangiogenesis processes (12, 13). Furthermore, these patients have presented a systematic increase in malondialdehyde levels, correlated with a decrease in fetal pH in newborns (12). Despite this potential mechanism, we found no association of LEPVI with fetal distress. There could be two potential explanations for such finding: firstly, placental dysfunction due to chronic hypoxia tends to be corrected by increasing the number of villi (4). In addition, the fetus develops in an environment of hypoxia. Therefore, it is only in a greater need for physiological requirements for tissue homeostasis, when the inability to provide oxygen and nutrients could occur, with a risk to the well-being of the fetus. Secondly, when fetal distress is detected, a cesarean delivery is frequently performed. Therefore, we only expected a small number of cases in our database.

Because of the cross-sectional nature of the study, we cannot establish temporality. Moreover, it is possible that the presence of LEPVI in pregnant women is underreported. Therefore, our findings could be affected by non-differential misclassification bias, leading to an underestimation of the association observed. In addition, this database lacked information on prescriptions and treatments that may confound the results. However, we adjusted for comorbidities and for the presence of other risk factors related to lifestyle. Despite these adjustments, the lack of information in prescriptions or antenatal care may have biased our results depending on the relationship with the exposure and/or outcome. Despite these limitations, the use of the CMBD data shows numerous strengths, such as a large sample size, consistency in the coding of the variables, and potential for generalization of the findings to the entire Spanish population of women.

## Conclusions

Our findings suggest that varicose veins may be a novel and important clinical risk factor for fetal well-being and health. The association between the presence of varicose veins and the existence of placental dysfunction could be especially relevant in cases with high risk of hypo-oxygenation of the fetus, such as the presence of anemias, coagulopathy, heart failure, and smoking. Thus, the inclusion of the diagnosis of venous insufficiency should be considered as part of the monitoring protocol for pregnant women, as an external and easily detectable indicator of risk for placental dysfunction and IFC. Moreover, these findings call attention to the need for a prospective clinical study to establish the relevance of including LEVPI among the risk factors for IFC and its diagnosis, especially in low-risk populations.

## Data Availability Statement

The datasets presented in this study can be found in online repositories. The names of the repository/repositories and accession number(s) can be found in the article/Supplementary Material.

## Ethics Statement

This study was approved by the Clinical Research Ethics Committee of the Gómez-Ulla-UAH defense Hospital (37/17).

## Author Contributions

ÁA, CC, MO, NG-H, MÁ-M, LB, and JB contributed to data curation and formal analysis. ÁA, CC, MO, MÁ-M, JB, and LB wrote the manuscript. ÁA, CC, SC, LB, JD, MÁ-M, and LB did the investigation. ÁA, CC, MO, SC, MÁ-M, and JB helped with project administration and funding acquisition. All authors discussed the results and contributed to the final manuscript.

## Conflict of Interest

The authors declare that the research was conducted in the absence of any commercial or financial relationships that could be construed as a potential conflict of interest.
