# Supplementary material for: Association Between Lower Extremity Venous Insufficiency and Intrapartum Fetal Compromise: A Nationwide Cross-Sectional Study
Source: Front Med (Lausanne). 2021 Jul 9;8:577096. doi: 10.3389/fmed.2021.577096 (PMC8300430; doi:10.3389/fmed.2021.577096)
Supplement: Supplementary file 1 [file Table_1.DOCX]

**Supplementary material (online-only material)**

Table 1S: International Classification of Diseases, Ninth Revision, Clinical Modification (ICD 9 CM) codes used for the diagnosis

| **VARIABLE** | **CODES IDC9-CM (2014 REV)** |
| --- | --- |
| AIDS/HIV | 042x |
| Alcohol abuse | 265.2, 291.1-291.3,291.5-291.9, 303.0, 303.9, 305.0, 357.5, 425.5, 535.3, 571.0-571.3, 980.x, V11.3 |
| Anemia | 280.0-280.9, 281.x |
| Asthma | 493x |
| Cancer | 200.x-202.x, 203.0, 196.x-199.x, 140.x-195.x, |
| Cerebrovascular disease | 362.34, 430.x-438.x |
| Chronic pulmonary disease | 416.8, 416.9, 490.x--493x, 494x-505.x, 506.4, 508.1, 508.8 |
| Coagulopathy | 286.x, 287.1, 287.3-287.5 |
| Congestive heart failure | 398.91, 402.01, 402.11, 402.91, 404.01, 404.03, 404.11, 404.91, 404.93, 425.4-425.9, 428.x |
| Dementia | 290.x, 294.1, 331.2 |
| Depression | 296.2, 296.3, 296.5, 300.4, 309.0, 309.1, 301.12, 311 |
| Diabetes | 250.0- 250.9, 648.0, 648.8 |
| Drug abuse | 292.x, 304.x, 305.2-305.9, V65.42 |
| Hypertension | 401.x, 402.x-405.x 642.x |
| Hypothyroidism | 240.9, 243.x, 244.x, 246.1, 246.8 |
| Liver disease | 070.22, 070.23, 070.32, 070.33, 070.44, 070.54, 070.6, 070.9, 456.0-456.2, 570.x, 571.x, 572.2-572.8, 573.3, 573.4, 573.8, 573.9, V42.7 |
| Intrapartum fetal compromise | 656.3, 656.7, 656.8 |
| Multiple pregnancy | v27.2-v27.7 |
| Myocardial infarction | 410.x, 412.x |
| Newborn malformation (nm) | 655x , 656x , 740x-759x |
| Obesity | 278.0 |
| Paralysis | 334.1, 342.x, 343.x, 344.0-344.6, 344.9 |
| Peptic ulcer disease excluding bleeding | 531.x-534.x |
| Peripheral vascular disease | 093.0, 437.3, 440.x, 441.x, 443.1-443.9, 557.1, 557.9, V43.4 |
| Peripheral vascular disorders | 093.0, 437.3, 440.x, 441.x, 443.1-443.9, 447.1, 557.1, 557.9, V43.4 |
| Renal disease | 403.01, 403.11, 403.91, 404.02, 404.03, 404.12, 404.13, 404.92, 404.93,582x, 583.0-583.7, 585.x, 586.x, 588.0, V42.0, V45.1, V56.x |
| Rheumatic disease | 446.5, 710.0-710.4, 714.0-714.2, 714.8, 725.x |
| Smoke | 989.84, 649.0, 305.1, V15.82 |
| Varicose veins in the lower extremities or pelvis (LEPVI) | 671.0, 671.1, 671.8 |
